# Regional evaluation of childhood acute lymphoblastic leukemia genetic susceptibility loci among Japanese

**DOI:** 10.1038/s41598-017-19127-7

**Published:** 2018-01-15

**Authors:** Kevin Y. Urayama, Masatoshi Takagi, Takahisa Kawaguchi, Keitaro Matsuo, Yoichi Tanaka, Yoko Ayukawa, Yuki Arakawa, Daisuke Hasegawa, Yuki Yuza, Takashi Kaneko, Yasushi Noguchi, Yuichi Taneyama, Setsuo Ota, Takeshi Inukai, Masakatsu Yanagimachi, Dai Keino, Kazutoshi Koike, Daisuke Toyama, Yozo Nakazawa, Hidemitsu Kurosawa, Kozue Nakamura, Koichi Moriwaki, Hiroaki Goto, Yujin Sekinaka, Daisuke Morita, Motohiro Kato, Junko Takita, Toshihiro Tanaka, Johji Inazawa, Katsuyoshi Koh, Yasushi Ishida, Akira Ohara, Shuki Mizutani, Fumihiko Matsuda, Atsushi Manabe

**Affiliations:** 1 Department of Social Medicine, National Center for Child Health and Development, Tokyo, Japan; 2 Graduate School of Public Health, St. Luke’s International University, Tokyo, Japan; 3 Department of Pediatrics and Developmental Biology, Tokyo Medical and Dental University, Tokyo, Japan; 4 Center for Genomic Medicine, Kyoto University Graduate School of Medicine, Kyoto, Japan; 5 Division of Molecular and Clinical Epidemiology, Aichi Cancer Center Research Institute, Aichi, Japan; 6 Department of Clinical Pharmacy, Center for Clinical Pharmacy and Sciences, School of Pharmacy, Kitasato University, Tokyo, Japan; 7 Department of Hematology/Oncology, Saitama Children’s Medical Center, Saitama, Japan; 8 Department of Pediatrics, St. Luke’s International Hospital, Tokyo, Japan; 9 Department of Hematology/Oncology, Tokyo Metropolitan Children’s Medical Center, Tokyo, Japan; 10 Department of Pediatrics, Japanese Red Cross Narita Hospital, Chiba, Japan; 11 Department of Hematology/Oncology, Chiba Children’s Hospital, Chiba, Japan; 12 Department of Pediatrics, Teikyo University Chiba Medical Center, Chiba, Japan; 13 Department of Pediatrics, University of Yamanashi, Yamanashi, Japan; 14 Department of Pediatrics, Yokohama City University Graduate School of Medicine, Yokohama, Japan; 15 Department of Pediatrics, St. Marianna University School of Medicine, Kawasaki, Japan; 16 Division of Pediatric Hematology and Oncology, Ibaraki Children’s Hospital, Mito, Japan; 17 Division of Pediatrics, Showa University Fujigaoka Hospital, Yokohama, Japan; 18 Department of Pediatrics, Shinshu University School of Medicine, Matsumoto, Japan; 19 Department of Pediatrics, Dokkyo Medical University, Tochigi, Japan; 20 Department of Pediatrics, Teikyo University Hospital, Tokyo, Japan; 21 Department of Pediatrics, Saitama Medical Center, Saitama Medical University, Saitama, Japan; 22 Division of Hematology/Oncology & Regenerative Medicine, Kanagawa Children’s Medical Center, Yokohama, Japan; 23 Department of Pediatrics, National Defense Medical College, Saitama, Japan; 24 Children’s Cancer Center, National Center for Child Health and Development, Tokyo, Japan; 25 Department of Pediatrics, The University of Tokyo Hospital, Tokyo, Japan; 26 Department of Human Genetics and Disease Diversity, Tokyo Medical Dental University, Tokyo, Japan; 27 Bioresource Research Center, Tokyo Medical and Dental University, Tokyo, Japan; 28 Pediatric Medical Center, Ehime Prefectural Central Hospital, Matsuyama, Japan; 29 Department of Pediatrics, Toho University, Tokyo, Japan

## Abstract

Genome-wide association studies (GWAS) performed mostly in populations of European and Hispanic ancestry have confirmed an inherited genetic basis for childhood acute lymphoblastic leukemia (ALL), but these associations are less clear in other races/ethnicities. DNA samples from ALL patients (aged 0–19 years) previously enrolled onto a Tokyo Children’s Cancer Study Group trial were collected during 2013–2015, and underwent single nucleotide polymorphism (SNP) microarray genotyping resulting in 527 B-cell ALL for analysis. Cases and control data for 3,882 samples from the Nagahama Study Group and Aichi Cancer Center Study were combined, and association analyses across 10 previous GWAS-identified regions were performed after targeted SNP imputation. Linkage disequilibrium (LD) patterns in Japanese and other populations were evaluated using the varLD score based on 1000 Genomes data. Risk associations for *ARID5B* (rs10821936, OR = 1.84, *P* = 6 × 10^−17^) and *PIP4K*2*A* (rs7088318, OR = 0.76, *P* = 2 × 10^−4^) directly transferred to Japanese, and the *IKZF1* association was detected by an alternate SNP (rs1451367, OR = 1.52, *P* = 2 × 10^−6^). Marked regional LD differences between Japanese and Europeans was observed for most of the remaining loci for which associations did not transfer, including *CEBPE*, *CDKN2A*, *CDKN2B*, and *ELK3*. This study represents a first step towards characterizing the role of genetic susceptibility in childhood ALL risk in Japanese.

## Introduction

Scrutiny of the human genome through evaluation of common genetic variants has revealed hundreds of disease susceptibility loci. In childhood acute lymphoblastic leukemia (ALL), six regions that have replicated in several populations are now considered known susceptibility loci (likely representing associations with *ARID5B*, *IKZF1*, *CEBPE, CDKN2A*, *PIP4K2A*, and *GATA3*), with the majority of the evidence supported through studies conducted in populations of European and Hispanic descent^1,2^. Gains in statistical power achieved by recent meta-analyses of childhood ALL genome-wide association studies (GWAS) have resulted in the identification of risk-associated single nucleotide polymorphisms (SNPs) of comparatively lower allele frequencies and estimated magnitude of effects including those tagging the *CDKN2B*, *LHPP*, and *ELK3* genes^3,4^. Furthermore, the concept that genetic susceptibility studies may potentially reveal race/ethnicity-specific associations was demonstrated by a recent GWAS conducted in a Chinese population which implicated a role for the *WWOX* gene that had not been observed in the numerous previous studies conducted in populations of European and Hispanic ancestry^5^.

Success of GWAS in identifying true disease-associated loci have largely been due to the consistently high standards in methodological rigor of the approach including, strict quality control for genotype data, attention to issues of statistical power and sample size, criteria for genome-wide significance, and integrating components of independent validation and/or functional evaluation of loci^6^. However, as is the case with other complex diseases, it is well-recognized that the known GWAS ‘hits’ in childhood ALL account for only a small proportion of the total estimated heritability^7^. Based on data from populations of European ancestry, it has been estimated that the currently known childhood ALL associated risk loci account for about 19 percent of the additive heritable risk, not accounting for potential impact of epistasis or gene-environment interactions^4^. Adding to this issue of ‘missing heritability’ is the realization that we currently know even less about the nature of childhood ALL genetic susceptibility in other populations, particularly Asians^8^.

The effects of known genetic susceptibility loci have yet to be fully confirmed in populations of non-European ancestry. Targeted validation attempts based on the same SNPs originally identified in mostly non-Hispanic whites and Hispanics have been performed in Chinese and other Asian populations, but findings have been inconsistent^9,10^. Assuming the same causal variant is operative across populations, a lack of association in Asians can be attributed to study flaws, but more likely due to reduced statistical power as a result of differences in allele frequency, strength of linkage disequilibrium (LD) with the causal variant(s), and/or the role of environmental exposures in affecting risk. Thus, a comprehensive characterization of genetic variation across the targeted genetic loci is required for an appropriate validation attempt in different populations.

To address this current gap in knowledge, we initiated an effort through the Tokyo Children’s Cancer Study Group (TCCSG) to assemble resources for genomic investigation of germline contributions to childhood ALL susceptibility and outcomes. Here, we report results of our targeted analysis of previous GWAS-identified childhood ALL risk loci in a large Japanese population. We evaluated the transferability of risk associations of specific SNP loci in Japanese and interpreted the finding in the context of quantified differences in LD within those loci across populations. Furthermore, we analyzed regional SNP data in order to identify alternate SNPs which may potentially confer stronger association in Japanese.

## Materials and Methods

### Study Population

In collaboration with a large network of 23 hospitals participating in the Tokyo Children’s Cancer Study Group (TCCSG), previously diagnosed childhood ALL patients visiting for a routine follow-up between 2013 and 2015 were invited to participate in this study. The TCCSG network includes nearly all clinical centers that diagnose and treat childhood ALL within the seven prefectures that comprise the Kanto and immediately surrounding regions^11,12^. Patients were considered eligible if they were 19 years of age or younger at the time of ALL diagnosis, enrolled onto a TCCSG treatment protocol, and self-identified as Japanese. Due to the nature of this sampling scheme, the study population comprised a survivorship population of childhood ALL patients.

Upon obtaining written informed consent, saliva samples using the Oragene Saliva DNA Self-Collection Kit (4 years of age and older) or Assisted Collection Kit (less than 4 years of age) (DNA Genotek, Ottawa, Canada) were collected from the patients with instruction by the attending physician or nurse during the follow-up outpatient visit. The collected samples were shipped at room temperature to a central laboratory (Tokyo Medical and Dental University) for processing, DNA extraction, and storage.

Controls comprised a subset of adult participants enrolled in two ongoing epidemiological studies of lifestyle-related chronic diseases in Japan, the Nagahama Study Group^13^ and Aichi Cancer Center Study^14^, in which large-scale genome-wide SNP genotyping had already been performed. The Nagahama Study is a community-based prospective cohort study comprising a representative sample of residents of Nagahama City in Shiga Japan^15^. The Aichi Cancer Center Study comprised a hospital-based cohort of non-cancer outpatient visitors^16^. Despite the name, the Aichi Cancer Center resembles a general hospital that does not require physician referral in which the majority of outpatients present with no abnormal findings by clinical examination. Population substructure across regions of Japan does exist; most notably between populations of Okinawa and the other main islands of Japan collectively. Although cases and controls were recruited from different regions of the main island, simulation studies have shown only minimal genomic inflation potential when considering these two subpopulations^17^. A history of childhood leukemia was not assessed in controls; however, the rarity of this disease suggests that any previous diagnosis of childhood leukemia in controls would have a minimal effect on the results of this study.

This study protocol was approved by the institutional review boards of St. Luke’s International Hospital, Tokyo Medical and Dental University, Kyoto University, and all collaborating hospitals involved in patient recruitment. Written informed consent was obtained from the parents of each participant together with a written assent by the child where possible. Patients aged 16 to 19 years were asked to provide written informed consent together with parental consent; those aged 20 or older did not require parental consent. This study was conducted in accordance with the Declaration of Helsinki.

### Genotyping and Quality Control

DNA extraction from childhood ALL patients’ saliva samples were performed using the Oragene prepIT DNA Extraction Kit (DNA Genotek) based on the manufacturer’s instruction. The approximately 2 mL saliva samples obtained from the Oragene Self-Collection Kits yielded, on average, a total of about 50 ug of genomic DNA.

Genome-wide SNP genotyping was attempted on 621 patient samples using the Illumina HumanCoreExome-12 v1.1 BeadChip (San Diego, CA) which contained probes for approximately 550,000 SNPs. Existing control data were genotyped previously using variable versions of the same Illumina HumanCoreExome BeadChip. Quality control steps were conducted within cases and each of the two different control sample series separately. SNPs were excluded if the genotype call rate was below 99%, the distribution of genotypes clearly deviated from that expected by Hardy-Weinberg equilibrium (HWE) (*P* < 1 × 10^−6^), or the minor allele frequency was less than 0.01. Samples were excluded if showed a genotyping success rate of less than 95% (51 cases and 4 controls) and relatedness based on an identity-by-descent analysis (1 case and 119 controls). In addition, principal components analysis (PCA) based on a genome-wide subset of SNPs in low LD (pruned at *r*^*2*^ < 0.1) that passed quality control steps was performed on a known ethnically homogeneous population of Japanese ancestry (International HapMap Project) together with cases and controls. The PCA was conducted using the EIGENSTRAT 2.0 software package and outlier samples were excluded (2 cases and 5 controls)^18^. In result, after quality control steps and excluding 40 T-cell ALL patients, the final population for analysis included a total of 527 Japanese B-cell ALL cases and 3,882 controls with data available for 171,547 SNPs that were overlapping across the genotyped case and control series.

Targeted SNP imputation was performed on the combined case-control dataset for 10 genomic regions reported in previous childhood ALL GWAS (Table 1) using ShapeIT2^19^ and Minimac3^20^, and the 1000 Genomes Project Phase III Version 5 as the reference population^21^. Poorly imputed SNPs defined by an R^2^ < 0.5 were excluded from the analyses. Considering the gene and its broad surrounding region (about 100-kb flanking) for each locus, a total of 113 SNPs were excluded among 14,457 total SNPs imputed across the 10 regions. On average, about 0.8 percent of SNPs per locus were excluded based on this quality control threshold. Due to restrictions stipulated by the institutional review board approvals, data were not be made publicly available, but may be available on request in compliance with the policies and procedures of the TCCSG.

**Table 1:** Previously identified genetic variants from genome-wide association studies and risk of childhood B-cell ALL in Japanese.

Gene/SNP^a^	Chr: Position^b^	Alleles	Cases (MAF) n = 527	Controls (MAF) n = 3,882	OR (95% CI)^c^	*P*
***ARID5B*** **(10q21.2)**						
rs10994982	10:63,710,104	G/A	0.583	0.466	1.58 (1.37–1.82)	3.83 × 10^−10^
rs10821936	10:63,723,577	T/C	0.500	0.345	1.84 (1.60–2.13)	6.04 × 10^−17^
rs7089424	10:63,752,159	T/G	0.489	0.344	1.76 (1.53–2.03)	7.77 × 10^−15^
***IKZF1*** **(7p12.2)**						
rs11978267	7:50,466,304	A/G	0.092	0.097	1.13 (0.89–1.44)	0.321
rs4132601	7:50,470,604	T/G	0.091	0.092	1.19 (0.93–1.51)	0.164
***CEBPE*** **(14q11.2)**						
rs4982731	14:23,585,333	T/C	0.136	0.133	1.11 (0.91–1.37)	0.306
rs2239633	14:23,589,057	G/A	0.452	0.467	0.93 (0.80–1.07)	0.282
***CDKN2A, CDKN2B*** **(9p21.3)**						
rs3731217	9:21,984,661	A/C	0.191	0.183	1.11 (0.93–1.33)	0.251
rs662463	9:22,030,438	G/A	0.011	0.013	0.85 (0.45–1.62)	0.625
rs17756311	9:22,053,895	G/A	0.010	0.012	0.88 (0.45–1.71)	0.695
***PIP4K2A*** **(10p12.2)**						
rs10828317	10:22,839,628	T/C	0.331	0.390	0.76 (0.65–0.88)	3.03 × 10^−4^
rs7088318	10:22,852,948	A/C	0.333	0.390	0.76 (0.65–0.88)	2.43 × 10^−4^
***GATA3*** **(10p14)**						
rs3824662	10:8,104,208	C/A	0.339	0.307	1.15 (1.00–1.33)	0.058
***LHPP*** **(10q26.13)**						
rs35837782	10:126,293,309	G/A	0.414	0.393	1.06 (0.92–1.22)	0.442
***ELK3*** **(12q23.1)**						
rs4762284	12:96,612,762	T/A	0.415	0.430	0.93 (0.80–1.07)	0.303
***WWOX*** **(16q23.1)**						
rs1121404	16:79,089,869	T/C	0.412	0.383	1.04 (0.90–1.19)	0.623

Abbreviations: Chr, chromosome; CI, confidence interval; MAF, minor allele frequency; OR, odds ratio; SNP, single nucleotide polymorphism.

^a^SNPs showing the strongest associations in previous genome-wide association studies (GWAS) within the identified region was selected. If multiple GWAS conducted in the same racial/ethnic population reported different SNPs, but tagged the same genomic region, SNPs from the first report were selected.

^b^Genomic positions are based on the human genome assembly GRCh37 coordinates.

^c^Odds ratios and 95% confidence intervals were calculated using logistic regression assuming a log-additive genetic model and adjusting for 10 PCA eigenvectors.

### Statistical Analysis

We first tested the association between childhood ALL and 16 SNPs across the 10 genes (Table 1) identified in previous GWAS. SNPs for evaluation were selected based on the strongest result reported from the first study to report the association. Multiple SNPs tagging the same genomic region were selected if the SNP was examined across several studies. We examined the role of additional genetic variation across the entire span of the 10 targeted genes, including a 10-kb flanking region on both ends. The association between each genetic variant and risk of childhood ALL was estimated by the odds ratio (OR) per allele and 95% confidence intervals (CI) using multiple logistic regression assuming a log-additive genetic model. Genome-wide association analysis of the 171,547 SNPs showed evidence of genomic inflation (λ > 1.10); all analyses were adjusted for 10 PCA eigenvectors (λ = 1.05). For the test of specific previously reported GWAS SNPs, a nominal p-value of less than 0.05 was considered statistically significant. For the examination of other potentially associated SNPs across the genomic regions, to account for multiple comparisons in the presence of LD between SNPs, we calculated adjusted p-values based on 10,000 permutations of case-control status and considered p-values below a family-wise type I error rate threshold of 0.05 to be statistically significant. Analyses were conducted using PLINK^22^ and SAS software version 9 (SAS, Cary, NC). The LocusZoom web-based resource was used to generate plots of association results by genomic region^23^.

Differences across race/ethnic populations in regional patterns of LD flanking a 10-kb region on both ends of the SNPs were quantified using the variation in LD (varLD) score applied to the 1000 Genomes Phase 3 data^21^. The varLD score is an algorithm based on comparing regional patterns of correlation previously developed by Teo *et al*. to quantify differences in LD within defined regions^24,25^. With the exception of the *WWOX* locus, the Japanese (JPT) population was compared to the combined population of European ancestry (EUR); for the *WWOX* locus, JPT was compared to the combined Han Chinese and Southern Han Chinese (CHB-CHS) representing the population in which the locus was originally identified. Permutation procedures were performed to determine Monte Carlo statistical significance by comparing the estimated varLD score to the null distribution of varLD scores after successive re-sampling of the two populations from the combined data^25^; 10,000 iterations were performed. Since 9 genomic loci were tested (*CDKN2A*-*CDKN2B* were evaluated as one region), an empirical p-value of less than 0.006 was considered statistically significant for the varLD evaluation. All statistical tests were two-sided.

## Results

Association analyses were performed on a total of 527 B-cell ALL cases and 3,882 controls. Median age at ALL diagnosis was 4.5 years (range: 0.3–16.8 years). The risk of childhood B-cell ALL associated with 16 SNPs (representing 10 genes) reported in previous GWAS was evaluated in this Japanese population (Table 1). The *ARID5B* SNPs showed strong evidence of an association with the highest risk observed for rs10821936 (OR = 1.84, 95% CI = 1.60–2.13, *P* = 6.04 × 10^−17^). Of the remaining loci, the 2 correlated *PIP4K2A* SNPs evaluated showed an association with childhood ALL (rs10828317, OR = 0.76, 95% CI = 0.65–0.88, *P* = 3.03 × 10^−4^) as well. The *GATA3* rs3824662 association was only suggestive (OR = 1.15, 95% CI = 1.00–1.33, *P* = 0.058), but was further supported by the presence of a nearby SNP in LD (rs2275806, *r*^*2*^ = 0.72) that showed a stronger association (OR = 1.20, 95% CI = 1.04–1.38, *P* = 0.011). The *WWOX* SNP, rs1121404, recently identified to be associated with childhood ALL in Chinese, showed no association in Japanese (OR = 1.04, 95% CI = 0.90–1.19, *P* = 0.623).

Risk allele frequencies and association estimates across various races/ethnicities are presented in Table 2. Among the loci identified through GWAS, only *ARID5B* SNPs showed a consistent association across the race/ethnic populations despite marked differences in allele frequencies. Although only marginally significant in Chinese (rs7088318, OR = 1.23, *P* = 0.047), the *PIP4K2A* association also showed consistency across populations. Primary SNPs first reported in populations of European ancestry for *IKZF1* (rs4132601 and rs11978267), then subsequently replicated in Hispanics and African Americans, showed no association in both Chinese and Japanese. The risk allele frequencies for the SNPs in Japanese (approximately 0.10) are markedly lower than frequencies observed in the original GWAS populations (approximately 0.20–0.30). Risk-associated SNPs recently identified in *LHPP* and *ELK3* in Europeans have not yet been reported in other populations. In Japanese, rs35837782 in *LHPP* and rs4762284 in *ELK3* did not show an association.

**Table 2:** Summary of genetic variants and childhood ALL risk associations across races/ethnicities identified through genome-wide association studies.

Gene/SNP	European Ancestry				Hispanic Ancestry				African Ancestry				Chinese Ancestry			Japanese Ancestry^d^		
	RAF^a^	OR^b^	*P*	Ref	RAF^a^	OR^b^	*P*	Ref	RAF^a^	OR^b^	*P*	Ref	RAF^a^	OR^b^	*P*	RAF^a^	OR^b^	*P*
***ARID5B***																		
rs10821936	0.33	1.91	1 × 10^−15^	^27^	0.47	1.95	4 × 10^−11^	^42^	0.24	1.52	4 × 10^−3^	^42^	0.38	1.43	5 × 10^−4^	0.35	1.84	6 × 10^−17^
***IKZF1***																		
rs4132601	0.28	1.69	1 × 10^−19^	^28^	0.27	1.46	1 × 10^−3^	^32^	—	—	—	—	0.14	1.20	0.197	0.09	1.19	0.164
rs11978267	0.27	1.69	9 × 10^−11^	^27^	0.26	1.31	0.01	^42^	0.19	1.59	5 × 10^−3^	^42^	—	—	—	0.10	1.13	0.321
***CEBPE***																		
rs2239633	0.52	1.34	2.9 × 10^−7^	^28^	0.61	1.35	6.6 × 10^−3^	^32^	—	—	—	—	0.64	1.18	0.117	0.53	1.08	0.282
rs4982731	0.28	1.29	9.1 × 10^−6^	^42^	0.39	1.58	2.3 × 10^−6^	^42^	0.38	1.13	0.410	^42^	—	—	—	0.13	1.11	0.306
***CDKN2A, CDKN2B***																		
rs3731217	0.86	1.41	3 × 10^−11^	^34^	0.88^c^	1.76	5 × 10^−3^	^32^	—	—	—	—	0.80	1.04	0.769	0.82	0.90	0.251
rs662463	0.10	1.48	2 × 10^−10^	^3^	0.07	1.45	0.034	^3^	0.12	1.55	3 × 10^−3^	^3^	—	—	—	0.01	0.85	0.625
rs17756311	0.09	1.43	3 × 10^−5^	^42^	0.06	1.36	0.100	^42^	0.10	1.12	0.620	^42^	—	—	—	0.01	0.88	0.695
***PIP4K2A***																		
rs7088318	0.59	1.25	5 × 10^−6^	^42^	0.75	1.42	9 × 10^−3^	^42^	0.39	1.65	1 × 10^−3^	^42^	0.58	1.23	0.047	0.61	1.32	2 × 10^−4^
***GATA3***																		
rs3824662	0.17	1.31	9 × 10^−12^	^40^	—	1.23	0.046	^43^	—	—	—	—	0.32	1.32	0.013	0.31	1.15	0.058
***LHPP***																		
rs35837782	0.62	1.21	1 × 10^−11^	^4^	—	—	—	—	—	—	—	—	—	—	—	0.61	0.94	0.442
***ELK3***																		
rs4762284	0.29	1.19	8 × 10^−9^	^4^	—	—	—	—	—	—	—	—	—	—	—	0.57	1.08	0.303
***WWOX***																		
rs1121404	—	—	—	—	—	—	—	—	—	—	—	—	0.28	1.38	5 × 10^−10^	0.38	1.04	0.623

Abbreviations: RAF, risk allele frequency; OR, odds ratio; Ref, reference; SNP, single nucleotide polymorphism.

^a^Frequency of the allele in controls conferring an increased risk of childhood ALL.

^b^Odds ratios indicate the risk associated with the each additive increase in risk conferring allele.

^c^Risk allele frequency obtained from the Human Genome Diversity Project (HGDP)

^d^Calculations pertain to the designated risk allele indicated in the primary report. Risk estimate results may not be greater than 1.0 in East Asians.

**Figure 1:**
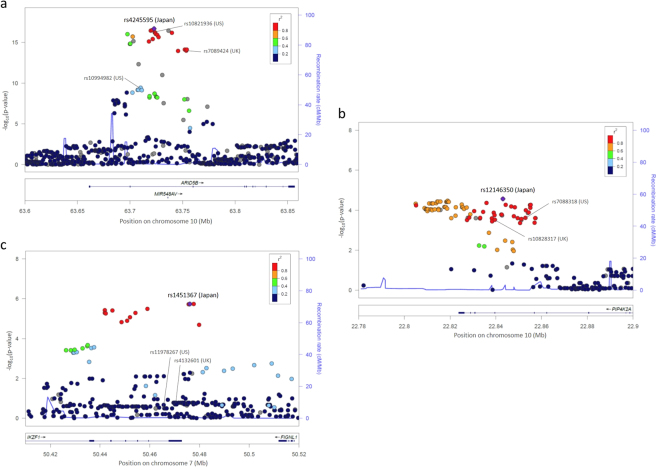
Regional plot of results of the association between SNPs in (**a**) *ARID5B*, (**b**) *PIP4K2A*, and (**c**) *IKZF1* and risk of childhood B-cell ALL. Multiple logistic regression was performed assuming a log-additive genetic model and adjusting for 10 principal components eigenvectors. The −log10 (p*-*value) for each SNP are plotted against their chromosomal position. The purple diamond **(♦)** indicates the strongest associated SNP in the region and the colors of the dots **(•)** represent the degree of linkage disequilibrium (based on *r*^2^) in relation to that index SNP in Japanese. Recombination rates (cM/Mb) overlay the plots. Coordinates are based on human genome assembly GRCh37 build.

Using available SNP data across all 10 genetic loci including 10-kb flanking regions on both ends of the target genes, B-cell ALL risk associations were identified for alternate SNPs in *IKZF1* (rs1451367, OR = 1.52, 95% CI = 1.28–1.80, *P* = 1.9 × 10^−6^) (Table 3 and Fig. 1). For the two genetic loci where the SNP associations directly transferred to Japanese, rs4245595 in *ARID5B* (OR = 1.86, *P* = 2.1 × 10^−17^) and rs12146350 in *PIP4K2A* (OR = 0.72, *P* = 2.7 × 10^−5^) showed slightly stronger p-values, and both were in strong LD (*r*^*2*^ > 0.90) in Japanese with the originally reported respective SNPs. The rs4245595 *ARID5B* SNP is also in strong LD (*r*^*2*^ > 0.96) with the recently reported functional SNP, rs7090445 (OR = 1.85, *P* = 3.1 × 10^−17^), identified by Studd *et al*. in which they showed influences on enhancer activity and *RUNX3* binding^26^. For the remaining genetic loci, alternate SNPs with a nominal p-values of less than 0.05 were identified, but were not statistically significant after adjustment for the number of SNPs tested across the respective regions.

**Table 3:** Comparison of linkage disequilibrium of ALL-associated genomic regions between populations of European and Japanese ancestry and alternate SNP associations in Japanese.

Locus	VarLD Evaluation (EUR vs JPN)^a^			Alternate SNP Association in Japanese within Gene						
	GWAS SNPs	Region (10-kb flanking)^b^	P_adj_^c^	SNP (allele)	Position	Ca-MAF	Co-MAF	OR^d^	P_nominal_	P_adj_^e^
**Loci associated in Japanese**										
*ARID5B*	rs10994982, rs10821936, rs7089424	63,700,104–63,762,159	0.031	rs4245595 (T/C)	63,722,895	0.50	0.35	1.86	2 × 10^−17^	9 × 10^−5^
*IKZF1*	rs11978267, rs4132601	50,456,304–50,480,604	0.018	rs1451367 (C/T)	50,477,661	0.23	0.17	1.52	2 × 10^−6^	4 × 10^−4^
*PIP4K2A*	rs10828317, rs7088318	22,829,628–22,862,948	0.109	rs12146350 (G/C)	22,843,111	0.32	0.39	0.72	3 × 10^−5^	2 × 10^−3^
**Association uncertain**										
*CEBPE*	rs4982731, rs2239633	23,575,333–23,599,057	1 × 10^−4^	rs4981457 (G/A)	23,578,089	0.36	0.39	0.86	0.041	0.640
*CDKN2A/B*	rs3731217, rs17756311	21,974,661–22,063,895	1 × 10^−4^	9:21986535 (A/G)	21,986,535	0.04	0.03	1.28	0.169	0.965
*GATA3*	rs3824662	8,094,208–8,114,208	1 × 10^−4^	rs2275806 (A/G)	8,095,340	0.41	0.36	1.20	0.011	0.310
*LHPP*	rs35837782	126,283,309–126,303,309	0.053	rs113148868 (G/A)	126,275,824	0.21	0.18	1.24	0.014	0.862
*ELK3*	rs4762284	96,602,762–96,622,762	8 × 10^−3^	rs2075362 (A/G)	96,606,889	0.38	0.41	0.90	0.145	0.997
*WWOX* ^a^	rs1121404	79,079,869–79,099,869	0.187	rs2738652 (A/T)	78,494,356	0.37	0.42	0.78	6 × 10^−4^	0.617

Abbreviations: Ca, cases; Co, controls; EUR, European ancestry; GWAS, genome-wide association study; JPN, Japanese ancestry; kb, kilobase; MAF, minor allele frequency; OR, odds ratio; SNP, single nucleotide polymorphism; varLD, variation in linkage disequilibrium.

^a^Using 1000 Genomes Project Phase 3 data, regional LD patterns in Japanese were compared to patterns in the population for which the SNP association was first reported. *WWOX* varLD evaluations were performed compared to the Han Chinese population.

^b^Regions included the evaluated SNPs and an additional 10-kb span flanking both ends. Genomic positions are based on the human genome assembly GRCh37 coordinates.

^c^Adjusted p-values (P_adj_) were based on 10,000 permutations of the combined data comprising the two populations being compared. An adjusted p-value of less than 0.006 based on a Bonferroni correction was considered statistically significant since 9 genomic regions were being tested.

^d^Odds ratios indicate the risk associated with the each additive increase in minor allele. ^e^Adjusted p-values (P_adj_) were based on 10,000 permutations of case-control status and considered p-values below a family-wise type I error rate threshold of 0.05 to be statistically significant.

To examine whether the non-transferability of association may be due to population differences in regional LD structure, varLD scores were calculated using 1000 Genomes Project Phase 3 data for Japanese, Han Chinese, and populations of European ancestry (Table 3). The regions surrounding the *ARID5B* and *PIP4K2A* SNPs, loci that directly replicated in Japanese, did not show statistically significant evidence of regional LD differences between populations of European ancestry and Japanese. Regions surrounding the *IKZF1* SNPs also showed minimal evidence of regional LD differences, but the previous GWAS-identified SNP associations did not directly transfer to Japanese. However, alternate statistically significant SNPs within *IKZF1* were identified (described above). With the exception of *LHPP* and *WWOX*, the four additional genetic loci in which the association did not transfer to Japanese showed strong evidence of regional LD structure differences based on varLD evaluations.

## Discussion

Aided by successful validation across multiple populations, the genome-wide association analysis approach has led to the identification of several genetic loci involved in childhood ALL risk^1,2^. However, there is still uncertainty about the role of these loci and consistency of specific SNP associations in East Asians with the majority of robust studies being performed primarily in populations of European and Hispanic ancestries. In our targeted evaluation of 16 previous GWAS-reported SNPs, we observed that the risk associations of those in *ARID5B* and *PIP4K2A* directly transfer to the Japanese population. The involvement of *IKZF1* is also supported by the identification of alternate associated SNPs in proximity to the originally reported loci, and the *GATA3* locus appears suggestive. However, this leaves the associations in the six remaining genes without clear evidence for a role in childhood ALL risk in East Asians. Examination of regional varLD scores showed that significant differences in LD between Japanese and the population in which the association was first reported were commonly observed in genes where the risk association did not transfer. Rather than concluding that the association is not present in Japanese, the varLD observations suggest that the associations may be obscured by differences in LD patterns and that other strategies are necessary to further clarify the role of the remaining six loci that did not transfer to this population.

Childhood ALL SNP associations in *ARID5B* first reported concurrently in studies performed in populations of European ancestry in the United States^27^ and the United Kingdom^28^ have been widely validated across multiple race/ethnic population^29^, now including Japanese. The risk-conferring minor allele frequency of rs10821936 in Japanese is similar to that of Europeans (MAF ~ 0.35), but is significantly higher in Hispanics (MAF ~ 0.45) and lower in populations of African ancestry (MAF ~ 0.20). Interestingly, this pattern is similar to the relative population differences in incidence of childhood ALL and evidence supports a role for this locus in partially explaining this difference. Based on available data from St. Jude Children’s Research Hospital and descriptive statistics from the Surveillance, Epidemiology, and End Results Program in the US, it was estimated that about 30% of the observed racial differences in ALL incidence may be attributable to the higher frequency of the rs10821936 risk allele in non-Hispanic whites compared to blacks^30^. Characterization of genetic ancestry of the Children’s Oncology Group Hispanic population showed increasing rs10821936 risk allele frequencies with increasing percentages of Native American ancestry^31^. Building on this observation, the California Childhood Leukemia Study reported increasing proportions of Native American ancestry to be associated with increasing risk of childhood ALL and showed that *ARID5B* contributes directly to the higher incidence in Hispanics compared to non-Hispanic whites^32^. However, the contribution of *ARID5B* is less clear in relation to Japanese given that this SNP has similar frequency and magnitude of effect as non-Hispanic whites despite known differences in incidence between the two populations.

Although consistently replicating in populations of European ancestry^9^, similar to studies performed in Chinese^5^, the *IKZF1* SNP association did not transfer to the Japanese population. Comparison of LD patterns based on varLD score between Europeans and Japanese did not show evidence of marked difference across an approximately 25-kb region comprising the previously reported SNPs. However, the allele frequency of the SNPs are considerably lower in East Asians at about 0.10 or less compared to close to 0.30 in Europeans and Hispanics. The ability to analyze the effect of other SNPs across the flanking regions led to the identification of an alternate associated SNP (rs1451367) located within about 10-kb that is common in East Asians (MAF ~ 0.20), but rare in Europeans (MAF < 0.01). This suggests that variation in *IKZF1* is also associated with risk of childhood ALL in Japanese; however, it cannot be concluded yet whether the SNP associations are representing the same causal locus across the populations.

Based on the results of the current analysis, evidence for childhood ALL risk associations with GWAS-identified SNPs in *CEBPE*, *CDKN2A*, *CDKN2B*, *LHPP*, *ELK*, and *WWOX* is lacking. Associations represented by other SNPs potentially tagging a causal locus within these genes were also not apparent. While the evidence is still limited, results could be influenced by differences in a gene-environment effect across populations not appropriately captured, or it may be possible that certain common SNPs identified in GWAS may be representing associations with rare causal variant(s) on the same haplotype background of the GWAS-identified tag SNP^33^. If rare causal variants are at play, even modest differences in haplotype structure of the regions may significantly affect detection potential, or it is possible that the variants may not be present in Japanese. As an example, the *CDKN2A* risk association originally identified through GWAS based on the common variant rs3731217^34^ was recently shown to be explained by a rare high-impact coding variant (rs3731249)^35–37^. This variant is present in about two percent of Europeans, but is not present in Japanese. With the exception of *LHPP*, all loci for which the associations did not transfer showed evidence of differences in genetic architecture between Japanese and Europeans based on varLD score, whereas those that transferred did not show marked differences. In line with the common disease/common variant hypothesis^38^, if the GWAS associations are instead tagging a common causal variant and assuming this variant is operative as a risk locus in Japanese as well, we would have expected the regional SNP coverage and statistical power of the current study to be sufficient to detect the association signal. The lack of association suggests a need for future studies to consider characterization of rare variants in order to fully understand the nature of these GWAS loci in Japanese.

Certain limitations inherent to this study may have also affected the results. Although our study was limited to B-cell lineage ALL similar to most previous GWAS, availability of molecular subtype data was incomplete for a large proportion of the patients. While heterogeneity by subtype in the magnitude of risk has been observed for several of the loci, effects exclusive to a specific subtype have not been clearly demonstrated and is likely not the reason for the lack of association observed. One exception may be the *GATA3* risk locus identified in a GWAS of Ph-like ALL^39^ and another study that observed the association specifically in non-hyperdiploid B-cell ALL that lack the *ETV6-RUNX1* fusion^40^. Results for the *GATA3* variant (rs3824662) in the current study were suggestive of an association among the total B-cell ALL series, but requires further evaluation in a subtype specific analysis for confirmation. Also, access to patients for recruitment into this study was through the outpatient mechanism which resulted in a study population of surviving patients. This may have led to over-representation of patients of certain disease profiles; however a 80 to 85 percent survival rate of childhood ALL, as reported by the TCCSG^41^, suggests that the effect may have been minimal since our objectives focused on validating known GWAS hits, those of which were originally identified using general ALL patient populations that comprised of the most common ALL subtypes (versus a sequencing-based design targeting rare subtypes of poor prognosis). Finally, our data included imputed genotypes to enhance the coverage of genetic variation across the targeted genomic regions. Despite stringent quality control measures and advances in imputation methodologies, uncertainty still exists and may have introduced non-differential misclassification of genotypes and a reduction in statistical power to detect associations.

In this targeted evaluation of SNPs across regions previously identified in GWAS of childhood ALL, we showed that variation in *ARID5B*, *IKZF1*, *PIP4K2A*, and possibly *GATA3* contribute to the genetic susceptibility of childhood B-cell ALL in Japanese. There is a need to account for population-specificity in producing accurate risk prediction estimates based on inherited genetic variation. Thus, this analysis serves as the first step towards characterizing the role of genetic variation in the susceptibility to childhood ALL in the Japanese population. Identification of potential novel loci, perhaps specific to the East Asian population or those more detectable due to enhanced LD with a causal locus and/or allele frequency differences, may be possible through a genome-wide association analysis after expansion of this population for increased statistical power.

## References

[1] Moriyama T, Relling MV, Yang JJ (2015). Inherited genetic variation in childhood acute lymphoblastic leukemia. Blood.

[2] Urayama KY, Chokkalingam AP, Manabe A, Mizutani S (2013). Current evidence for an inherited genetic basis of childhood acute lymphoblastic leukemia. Int J Hematol.

[3] Hungate EA (2016). A variant at 9p21.3 functionally implicates CDKN2B in paediatric B-cell precursor acute lymphoblastic leukaemia aetiology. Nat Commun.

[4] Vijayakrishnan, J. *et al*. A genome-wide association study identifies risk loci for childhood acute lymphoblastic leukemia at 10q26.13 and 12q23.1. *Leukemia*, 10.1038/leu.2016.271 (2016). 10.1038/leu.2016.271 PMC5336191 27694927

[5] Shi Y (2016). Identification of a novel susceptibility locus at 16q23.1 associated with childhood acute lymphoblastic leukemia in Han Chinese. Hum Mol Genet.

[6] Hunter DJ (2012). Lessons from genome-wide association studies for epidemiology. Epidemiology.

[7] Gibson G (2010). Hints of hidden heritability in GWAS. Nat Genet.

[8] Urayama KY, Manabe A (2014). Genomic evaluations of childhood acute lymphoblastic leukemia susceptibility across race/ethnicities. [Rinsho ketsueki] The Japanese Journal of Clinical Hematology.

[9] Dai YE, Tang L, Healy J, Sinnett D (2014). Contribution of polymorphisms in IKZF1 gene to childhood acute leukemia: a meta-analysis of 33 case-control studies. PLoS One.

[10] Sun J (2015). Association between CEBPE Variant and Childhood Acute Leukemia Risk: Evidence from a Meta-Analysis of 22 Studies. PLoS One.

[11] Ishida Y (2014). Secondary cancers among children with acute lymphoblastic leukaemia treated by the Tokyo Children’s Cancer Study Group protocols: a retrospective cohort study. Br J Haematol.

[12] Kato M (2017). Long-term outcome of 6-month maintenance chemotherapy for acute lymphoblastic leukemia in children. Leukemia.

[13] Muro S (2016). Relationship Among Chlamydia and Mycoplasma Pneumoniae Seropositivity, IKZF1 Genotype and Chronic Obstructive Pulmonary Disease in A General Japanese Population: The Nagahama Study. Medicine.

[14] Seow WJ (2017). Association between GWAS-identified lung adenocarcinoma susceptibility loci and EGFR mutations in never-smoking Asian women, and comparison with findings from Western populations. Hum Mol Genet.

[15] Izuhara Y (2016). Mouth breathing, another risk factor for asthma: the Nagahama Study. Allergy.

[16] Inoue M (2003). Epidemiology of pancreatic cancer in Japan: a nested case-control study from the Hospital-based Epidemiologic Research Program at Aichi Cancer Center (HERPACC). International journal of epidemiology.

[17] Yamaguchi-Kabata Y (2008). Japanese population structure, based on SNP genotypes from 7003 individuals compared to other ethnic groups: effects on population-based association studies. Am J Hum Genet.

[18] Price AL (2006). Principal components analysis corrects for stratification in genome-wide association studies. Nat Genet.

[19] O’Connell J (2016). Haplotype estimation for biobank-scale data sets. Nat Genet.

[20] Das S (2016). Next-generation genotype imputation service and methods. Nat Genet.

[21] 1000 Genomes Project Consortium. A global reference for human genetic variation. *Nature* **526**, 68–74, 10.1038/nature15393 (2015). 10.1038/nature15393 PMC4750478 26432245

[22] Purcell S (2007). PLINK: a tool set for whole-genome association and population-based linkage analyses. Am J Hum Genet.

[23] Pruim RJ (2010). LocusZoom: regional visualization of genome-wide association scan results. Bioinformatics.

[24] Ong RT, Teo Y (2010). Y. varLD: a program for quantifying variation in linkage disequilibrium patterns between populations. Bioinformatics.

[25] Teo YY (2009). Genome-wide comparisons of variation in linkage disequilibrium. Genome Res.

[26] Studd JB (2017). Genetic and regulatory mechanism of susceptibility to high-hyperdiploid acute lymphoblastic leukaemia at 10p21.2. Nat Commun.

[27] Trevino LR (2009). Germline genomic variants associated with childhood acute lymphoblastic leukemia. Nat Genet.

[28] Papaemmanuil E (2009). Loci on 7p12.2, 10q21.2 and 14q11.2 are associated with risk of childhood acute lymphoblastic leukemia. Nat Genet.

[29] Zeng H (2014). Associations between AT-rich interactive domain 5B gene polymorphisms and risk of childhood acute lymphoblastic leukemia: a meta-analysis. Asian Pac J Cancer Prev.

[30] Yang W (2010). ARID5B SNP rs10821936 is associated with risk of childhood acute lymphoblastic leukemia in blacks and contributes to racial differences in leukemia incidence. Leukemia.

[31] Xu H (2012). ARID5B genetic polymorphisms contribute to racial disparities in the incidence and treatment outcome of childhood acute lymphoblastic leukemia. J Clin Oncol.

[32] Walsh KM (2013). Associations between genome-wide Native American ancestry, known risk alleles and B-cell ALL risk in Hispanic children. Leukemia.

[33] Wang K (2010). Interpretation of association signals and identification of causal variants from genome-wide association studies. Am J Hum Genet.

[34] Sherborne AL (2010). Variation in CDKN2A at 9p21.3 influences childhood acute lymphoblastic leukemia risk. Nat Genet.

[35] Vijayakrishnan J (2015). The 9p21.3 risk of childhood acute lymphoblastic leukaemia is explained by a rare high-impact variant in CDKN2A. Sci Rep.

[36] Walsh KM (2015). A Heritable Missense Polymorphism in CDKN2A Confers Strong Risk of Childhood Acute Lymphoblastic Leukemia and Is Preferentially Selected during Clonal Evolution. Cancer Res.

[37] Xu H (2015). Inherited coding variants at the CDKN2A locus influence susceptibility to acute lymphoblastic leukaemia in children. Nat Commun.

[38] Chakravarti A (1999). Population genetics–making sense out of sequence. Nat Genet.

[39] Perez-Andreu V (2013). Inherited GATA3 variants are associated with Ph-like childhood acute lymphoblastic leukemia and risk of relapse. Nat Genet.

[40] Migliorini G (2013). Variation at 10p12.2 and 10p14 influences risk of childhood B-cell acute lymphoblastic leukemia and phenotype. Blood.

[41] Tsuchida M (2010). Long-term results of Tokyo Children’s Cancer Study Group trials for childhood acute lymphoblastic leukemia, 1984–1999. Leukemia.

[42] Xu H (2013). Novel susceptibility variants at 10p12.31-12.2 for childhood acute lymphoblastic leukemia in ethnically diverse populations. J Natl Cancer Inst.

[43] Walsh KM (2013). GATA3 risk alleles are associated with ancestral components in Hispanic children with ALL. Blood.

